# Baseline Imaging Characteristics of Participants in the Phase II/III DIAN‐TU‐001 Tau NexGen Trial for Dominantly Inherited Alzheimer’s Disease

**DOI:** 10.1002/alz70859_105301

**Published:** 2025-12-25

**Authors:** Tammie L.S. Benzinger, Arnaud Charil, Brian A. Gordon, Shaney Flores, Russ C. Hornbeck, Kristin R Wildsmith, Sibabrata Banerjee, Guoqiao Wang, Eric McDade, Larisa Reyderman, Randall J. Bateman

**Affiliations:** ^1^ Washington University School of Medicine in St. Louis, St. Louis, MO USA; ^2^ Eisai Inc., Nutley, NJ USA; ^3^ Washington University School of Medicine, St. Louis, MO USA; ^4^ Washington University School of Medicine, St Louis, MO USA

## Abstract

**Background:**

The DIAN‐TU‐001 trial, conducted by the Dominantly Inherited Alzheimer Network Trials Unit (DIAN‐TU) at Washington University in St Louis, in collaboration with Eisai, is a study arm of the Tau NexGen platform study. This placebo‐controlled, double‐blind Phase II/III trial aims to evaluate the therapeutic effects of anti‐tau antibody etalanetug (E2814), administered alone or with lecanemab, on biomarkers, clinical efficacy, safety, and tolerability in participants with Dominantly Inherited Alzheimer Disease (DIAD). Here we report the baseline imaging characteristics of the trial population.

**Methods:**

Amyloid 11C‐PiB PET SUVr was converted to Centiloids, and both 18F‐MK6240 Tau SUVr and volumetric MRI (vMRI) values were calculated for Braak stage regions and eight composite regions of interest (ROIs).

Preliminary

**Results:**

The study includes symptomatic DIAD participants and asymptomatic mutation carriers. These groups differ in baseline amyloid levels (Symptomatic=99.86 (SD=39.45) CL; Asymptomatic=30.62 (SD=36.77) CL). Symptomatic subjects had nominally higher tau PET SUVr values across brain regions, with average values ranging from SUVR=1.94 in the hippocampus to 3.00 in the parietal cortex. Consistent with previous reports, early symptomatic DIAD subjects showed early tau accumulation in the parietal cortex, distinct from the pattern in early symptomatic sporadic AD subjects, who show initial tau accumulation in medial temporal regions (e.g., entorhinal cortex). Across the same tau PET composite ROIs, all vMRI values were nominally smaller in symptomatic vs asymptomatic subjects, reflecting atrophy. Higher atrophy in the symptomatic subjects is consistent with their higher tau PET SUVr values across all brain regions.

**Conclusion:**

The baseline imaging characteristics of the DIAN‐TU‐001 trial population reveal significant spatial differences in tau PET and vMRI between symptomatic and asymptomatic DIAD subjects. Baseline amyloid levels were consistent with the stage of disease in DIAD. Symptomatic DIAD subjects exhibit higher tau SUVR values and greater atrophy, particularly in the parietal cortex, compared to asymptomatic DIAD subjects. These tau PET findings corroborate the distinctly different pattern of tau deposition in DIAD vs. sporadic AD, as previously reported. Future analysis will elucidate the therapeutic effects of E2814 and lecanemab on this pathological tau spread.

